# Unraveling the Microbiome of Necrotizing Enterocolitis: Insights in Novel Microbial and Metabolomic Biomarkers

**DOI:** 10.1128/Spectrum.01176-21

**Published:** 2021-10-27

**Authors:** Chiara Tarracchini, Christian Milani, Giulia Longhi, Federico Fontana, Leonardo Mancabelli, Roberta Pintus, Gabriele Andrea Lugli, Giulia Alessandri, Rosaria Anzalone, Alice Viappiani, Francesca Turroni, Michele Mussap, Angelica Dessì, Flaminia Cesare Marincola, Antonio Noto, Anna De Magistris, Marine Vincent, Sergio Bernasconi, Jean-Charles Picaud, Vassilios Fanos, Marco Ventura

**Affiliations:** a Laboratory of Probiogenomics, Department of Chemistry, Life Sciences, and Environmental Sustainability, University of Parma, Parma, Italy; b Microbiome Research Hub, University of Parma, Parma, Italy; c GenProbio Srl, Parma, Italy; d Neonatal Intensive Care Unit, Department of Surgical Sciences, University of Cagliari, Monserrato, Italy; e Neonatal Intensive Care Unit, AOU, University of Cagliari, Cagliari, Italy; f Department of Chemical and Geological Sciences, University of Cagliari, Monserrato, Cagliari, Italy; g Department of Medical Science and Public Health, University of Cagliari, Monserrato, Cagliari, Italy; h Neonatology Unit, Croix-Rousse University Hospital, Hospices Civils de Lyon, Lyon, France; i CarMen laboratory, INSERM, INRA, Claude Bernard University Lyon, Pierre Benite, France; Emory University

**Keywords:** necrotizing enterocolitis, NEC, microbiota, metagenomics, shotgun

## Abstract

Necrotizing enterocolitis (NEC) is among the most relevant gastrointestinal diseases affecting mostly prematurely born infants with low birth weight. While intestinal dysbiosis has been proposed as one of the possible factors involved in NEC pathogenesis, the role of the gut microbiota remains poorly understood. In this study, the gut microbiota of preterm infants was explored to highlight differences in the composition between infants affected by NEC and infants prior to NEC development. A large-scale gut microbiome analysis was performed, including 47 shotgun sequencing data sets generated in the framework of this study, along with 124 retrieved from publicly available repositories. Meta-analysis led to the identification of preterm community state types (PT-CSTs), which recur in healthy controls and NEC infants. Such analyses revealed an overgrowth of a range of opportunistic microbial species accompanying the loss of gut microbial biodiversity in NEC subjects. Moreover, longitudinal insights into preterm infants prior to NEC development indicated Clostridium neonatale and Clostridium perfringens species as potential biomarkers for predictive early diagnosis of this disease. Furthermore, functional investigation of the enzymatic reaction profiles associated with pre-NEC condition suggested DL-lactate as a putative metabolic biomarker for early detection of NEC onset.

**IMPORTANCE** Necrotizing enterocolitis (NEC) is a severe gastrointestinal disease occurring predominantly in premature infants whose etiology is still not fully understood. In this study, the analysis of infant fecal samples through shotgun metagenomics approaches revealed a marked reduction of the intestinal (bio)diversity and an overgrowth of (opportunistic) pathogens associated with the NEC development. In particular, dissection of the infant’s gut microbiome before NEC diagnosis highlighted the potential involvement of *Clostridium* genus members in the progression of NEC. Remarkably, our analyses highlighted a gastrointestinal DL-lactate accumulation among NEC patients that might represent a novel potential functional biomarker for the early diagnosis of NEC.

## INTRODUCTION

Necrotizing enterocolitis (NEC) is a harmful gastrointestinal disease commonly encountered in neonatal intensive care units (NICU) worldwide. Upon NEC occurrence, segments of the infant’s gastrointestinal tract undergo ischemia and subsequently necrosis, thus representing a gastrointestinal emergency in neonatal age, occurring in about 8% of premature infants with a reported mortality rate of up to 25% (1). NEC is believed to be a disease with a multifactor etiology whose precise cause has not been fully understood. However, several risk factors have been identified. In particular, premature birth (less than 32 weeks of gestation) and very low birth weight (<1,500 g) have been reported to be among the main factors of increased risk of sepsis and NEC (1, 2). In addition, NEC has long been linked to microbial dysbiosis of the infant gut (3, 4). Indeed, it is well known that the first few days of life are a crucial time frame for the correct development and modulation of the human gut microbiota (5, 6). Therefore, inappropriate seeding of the microbial communities occurring at childbirth due to defects of optimal microbial acquisition, e.g., vertical mother-to-infant transmission, may have a short- or long-term impact on the host health (7–11). In this regard, it has been observed that an alteration of the taxonomic composition of the gut bacterial community and their functional properties characterize the gut microbiota of NEC patients compared with those of healthy infants (4, 12). More specifically, intestinal microbial communities of healthy breastfed infants are dominated by bifidobacterial species, mainly Bifidobacterium bifidum and Bifidobacterium longum subsp. *infantis* (6, 13). In contrast, the gut microbiota of NEC patients showed an increased abundance of *Clostridium* and *Enterobacteriaceae* genera (14, 15). In this context, an exacerbated proinflammatory cascade arising from dysfunctional, or overstated, immunological response to high levels of intestinal lipopolysaccharides (LPS) has been proposed as one possible pathway that predisposes the infant to NEC pathogenesis (16). Along with the above-mentioned direct host-microbe interactions mediated by the immune system, the gut microbiota was shown to exert a broad physiological effect on the host biochemistry through the gut microbiota metabolome, i.e., microbial-derived secondary metabolites. From this perspective, it becomes evident that perturbation of the microbial community composition may modify the intestinal metabolic profiling, with a subsequent impact on the host’s health. Overall, the proven importance of perinatal microbial exposures in health and illness provides the foundation for assuming that bacteria colonizing the infant gut in the immediate postnatal period may be involved in NEC development (17). Nevertheless, identifying specific causative microorganisms, known as microbial biomarkers, remains elusive (18).

In this study, we performed a metagenomics analysis of 171 preterm infant fecal samples, aiming to assess the infant gut microbiota composition during NEC events compared to those of gestational age-matched healthy infants. Furthermore, to identify putative microbial biomarkers of NEC, the obtained metagenomic data sets were also employed to determine the metabolic reactions profiles and the presence of potentially damaging microbial metabolites enriched in NEC.

## RESULTS AND DISCUSSION

### General features of data sets included in the meta-analysis.

A collection of 124 shotgun metagenomic data sets from four different studies (19–23) was retrieved from publicly available repositories (Table S1). Out of these, 67 corresponded to fecal samples of preterm infants suffering from NEC, while the remaining 57 samples were acquired from premature infants considered healthy overall. More precisely, among the NEC subjects, 53 were newborns with confirmed NEC at the time of sampling, while 14 fecal samples were collected prior to NEC diagnosis (Table S1). Gestational age ranged from 23 to 39 weeks, corresponding to a birth weight between 900 and 3,010 g. Notably, only four infants showed a gestational age or birth weight longer than 32 weeks and 1,500 g, respectively (Table S1). As gestational age, birth weight, and postnatal age are among the main factors strongly affecting the infant gut microbiota composition, the selection of only infants born very prematurely with low birth weight, and obtaining fecal samples from infants with a similar postnatal age, enabled us to establish a reliable approach for the comparison of the microbiota composition between these samples. However, according to what was shown previously, the gut microbiota seems to undergo only minor changes up to 6 months of age (24).

Additionally, 47 fecal samples of preterm infants from NICU at Croix Rousse University Hospital were also included in the analyses (Table S2). These samples were collected weekly during the first 30 days of life of 18 infants born between 25 and 30 weeks of gestation. While 11 infants did not display any intestinal morbidity (for a total of 24 fecal samples), 7 infants developed NEC, which was found in 12 fecal samples before and 11 fecal samples after NEC development and diagnosis (Table S2).

Overall, a total of 171 fecal samples of preterm infants, encompassing 64 cases of ongoing NEC, 26 patients that later established NEC but did not show symptoms at the time of collection, and 81 healthy control samples, were evaluated, representing one of the largest shotgun metagenomics data collections to date. All the data sets were reanalyzed using the same analysis pipeline, i.e., METAnnotatorX (25).

### Gut microbiota variability between cases of NEC and healthy subjects.

In order to highlight the differences in gut microbiota composition between infants with manifested NEC symptoms and healthy subjects, we compared the microbiota composition of the retrieved 64 NEC samples with the 81 healthy samples, while the 26 pre-NEC samples were investigated separately (see below). As generally expected for premature newborns, the index of bacterial species richness, i.e., biodiversity, calculated as the number of taxa with a relative abundance of sequenced reads greater than 0.5%, was on average relatively lower than that of term infants (26, 27) but still statistically higher in the healthy subjects (8.4 ± 5.7) than in those affected by NEC (6.6 ± 3.7, *t* test *P* < 0.05) (detailed data are reported in Table S3). This statistical reduction of the gut microbial biodiversity associated with NEC is supported by the observation that the two most abundant microbial species in the fecal samples of NEC patients cover 38% of the whole bacterial population, while those of the healthy infants cover 29.76%. Thus, this suggests that the loss of even few species may markedly disrupt the delicate ecological equilibrium established in the very early stages of life in the human gut environment.

The analysis of intersample variability of the gut microbiota composition revealed significant differences between infants with NEC diagnosis and control samples (permutational multivariate analysis of variance [PERMANOVA] *P* value of <0.05) (Fig. S1), reflecting the notion that the gut microbiome of newborns with NEC displayed not only lower biodiversity but also different taxonomic composition from that of their healthy counterparts (28).

For each metagenomic sample, shotgun methods allowed the taxonomic classification of bacterial taxa at the species level, with the estimation of their relative abundance (expressed as percentages of total sequenced reads per sample). Statistical identification of bacterial species with a relative differential abundance between the 81 controls and the 64 overt cases of NEC revealed that Escherichia coli and Enterococcus faecalis were the main taxa with statistically higher relative abundance in fecal samples of NEC patients, with an average abundance of 26.27% ± 41.06% and 11.24% ± 26.08%, respectively, compared to 13.85% ± 29.14% and 1.45% ± 4.06% in control samples, respectively (ANOVA *P* value of <0.01) (Table S3). In contrast, Streptococcus agalactiae was the dominant taxon in premature control subjects (average abundance of 15.91% ± 35.03% in comparison to 0.28% ± 1.77% within NEC population, ANOVA *P* value of <0.001) (Table S3), while both groups showed comparable levels of Staphylococcus epidermidis and Klebsiella pneumoniae, indicating at first glance that these bacterial taxa may not be directly related to NEC onset (Table S3).

However, the microbial profiles of the 145 collected infant gut metagenomic samples have markedly shown a high interindividual variability, suggesting that infant-specific factors and the different NICU environment may considerably affect the initial microbial colonization of the infants’ gut after birth, as reported previously (Table S3) (29, 30).

### Gut CSTs distribution in preterm infants.

**FIG 1:**
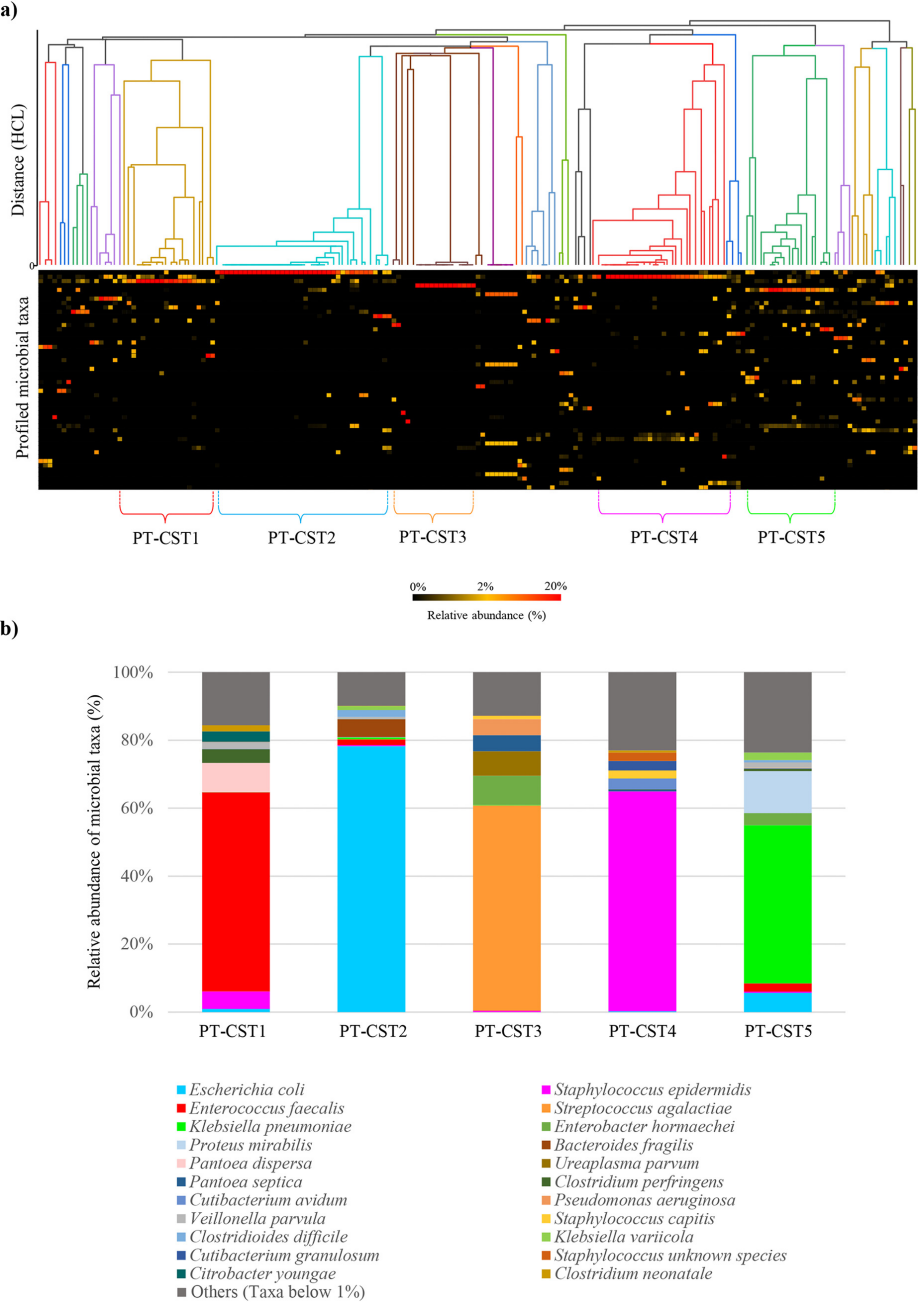
Identification of the five PT-CSTs. Panel a shows a cladogram of the 171 preterm infant fecal samples, obtained through hierarchical clustering (HCL) analysis. The cladogram highlights the five PT-CSTs identified through HCL analysis. Below is reported an overview of the taxonomic composition of the infant population. Panel b displays the average relative abundance of microbial species of the identified PT-CST, with relative abundances on the vertical axis and the sample on the horizontal axis.

The high taxonomic variability of the preterm gut microbial communities dramatically reduces the ability to detect significant microbial biomarkers. For this reason, we performed Pearson index-based hierarchical clustering analysis (HCA) employing species-level microbial profiling data to elucidate the prevalence of specific taxonomic patterns, also known as community state types (CSTs), across the collected samples (Table S4; Fig. 1a). The statistically validated clusters that encompassed at least 10 samples have been taken into account as the most prevalent representatives of preterm infants (24) (Table S4; Fig. 1a). Accordingly, we identified five archetypical subgroups named preterm community state types (PT-CST1 to 5) (Table S4; Fig. 1b) not correlated with specific geographical origins (Table S1). A detailed description of each PT-CST is provided in the supplementary text.

**FIG 2:**
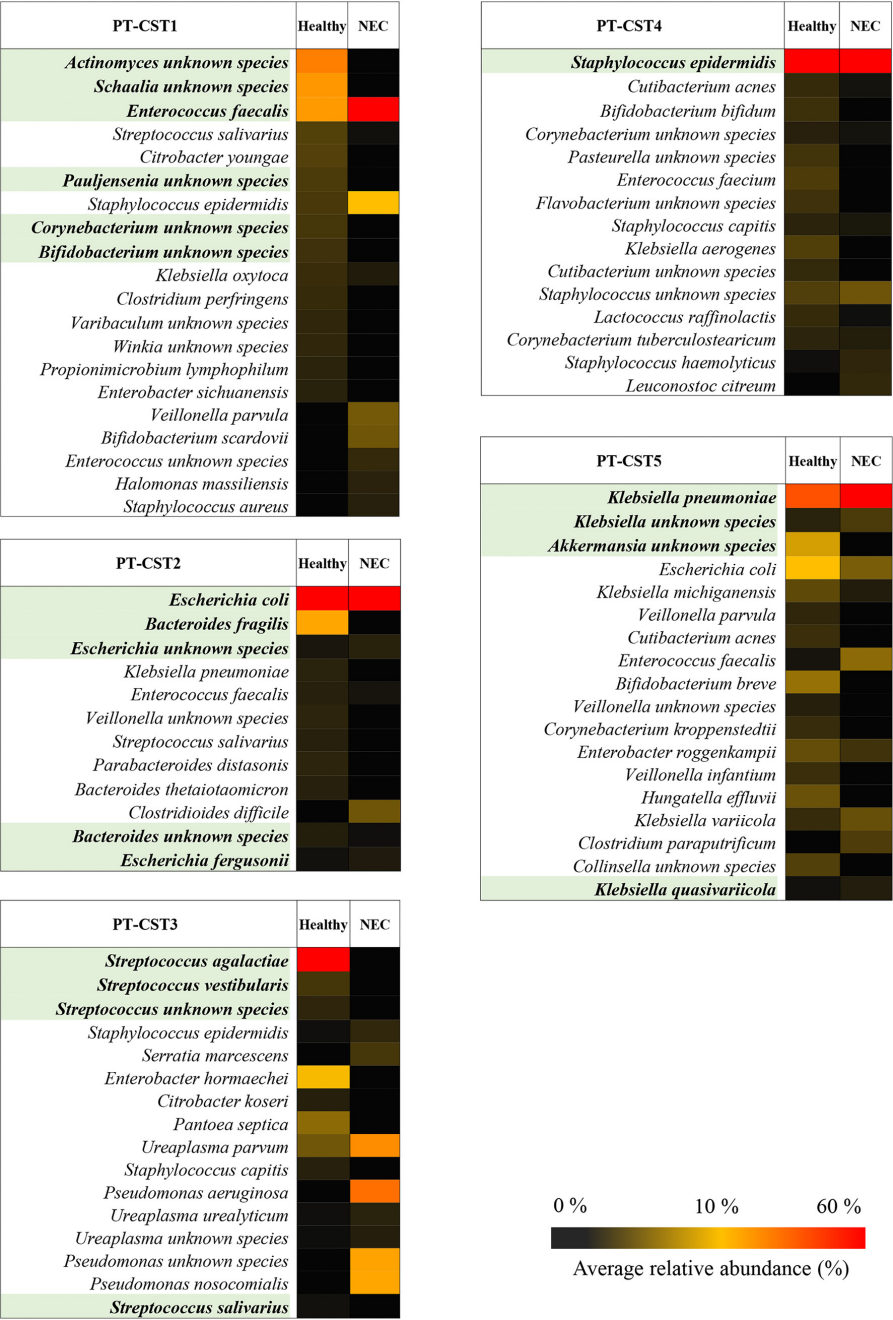
Statistically significant differences in the taxonomic composition between NEC and healthy samples of each PT-CST. In detail, for each PT-CST, species with significant *P* values obtained by comparison between microbial average abundances of healthy and NEC samples have been highlighted in green. Additional taxa above 1% of average abundance have been reported.

Notably, the five PT-CSTs were distributed unevenly across the fecal samples of healthy control and NEC preterm infants, thus revealing the absence of clear associations between specific taxonomic patterns and development of NEC (Table S4). Nevertheless, within each PT-CST (except for PT-CST3), a statistically significant reduction in biodiversity was observed in NEC rather than in control infants (species richness average ranged from 4.1 to 8.6 in NEC infants versus 7 to 19 in controls, *t* test *P* values of <0.01), accompanied by PT-CST-specific increase in relative abundances of well-known (opportunistic) pathogens, such as E. faecalis, E. coli, S. epidermidis, Clostridioides difficile, Ureaplasma parvum, Pseudomonas aeruginosa, Pseudomonas nosocomialis, and members of the Klebsiella genus (Table S5; Fig. 2). Notably, most of the latter species have been reported to exert pathogenic outcomes proportional to their abundance (31–38). These data suggest that the loss of specific taxa may play a direct role in the overgrowth of (opportunistic) pathogens supporting NEC development.

Detailed screening within each PT-CST for taxa involved in the loss of biodiversity revealed that a range of protective/health-promoting taxa appear reduced or absent in NEC subpopulations (Table S5; Fig. 2), including members of the genus *Bifidobacterium* (such as B. bifidum and Bifidobacterium breve) and *Akkermansia* (39–41). In addition, common early infant gut commensals, such as species of the genera *Actinomyces*, *Schaalia*, *Veillonella*, *Bacteroides*, and Streptococcus, appeared to be reduced or absent in the fecal samples of NEC patients (24, 42). Notably, since the latter are known to be among the early gut commensals, they may participate in the homeostasis of gut bacterial communities in preterm infants by acting as neutral commensals. Thus, their early loss, beyond causing the overall drop in biodiversity, probably supports the overgrowth of the taxa involved in NEC pathogenesis.

### Gut bacterial network community structure.

**FIG 3:**
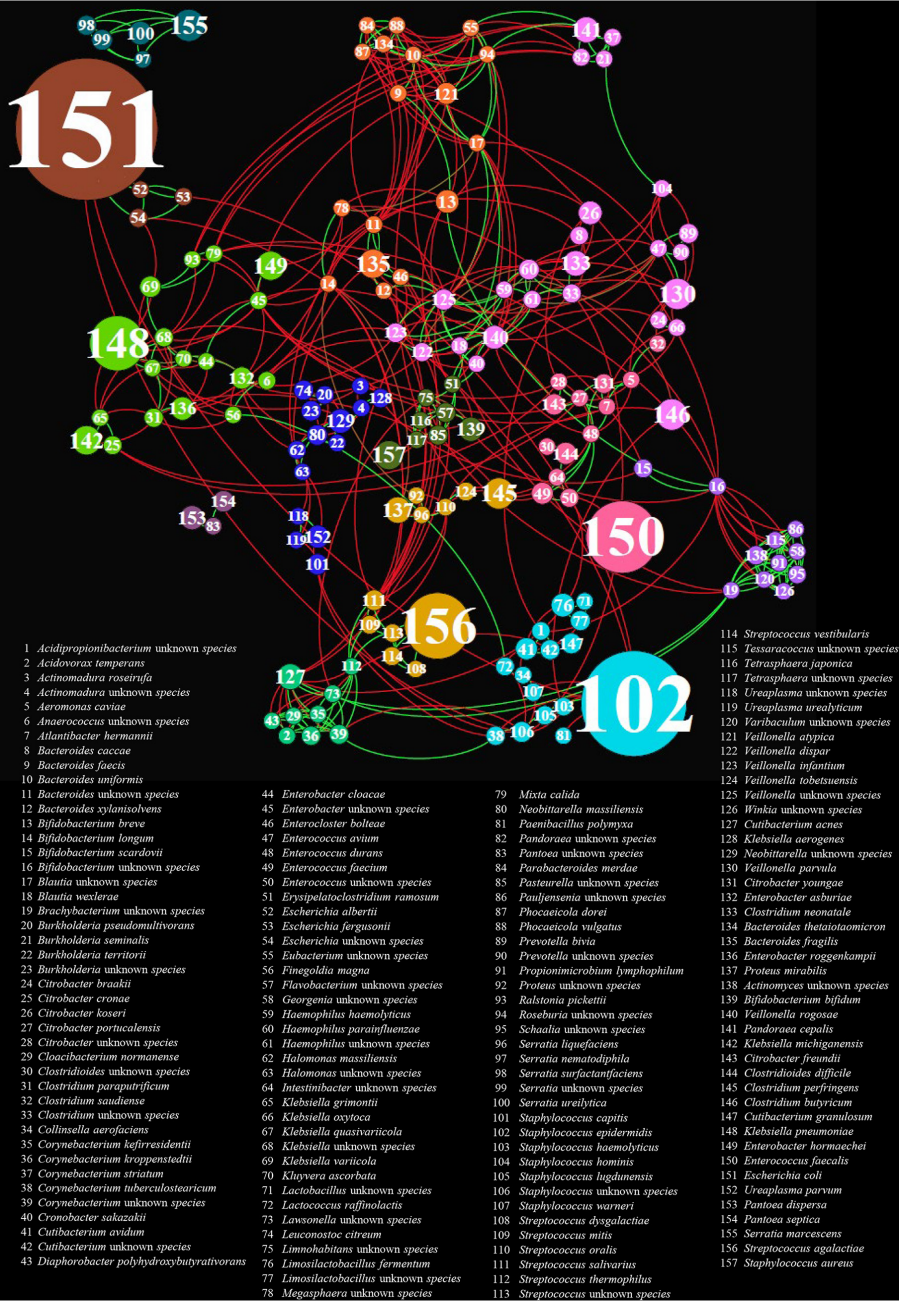
Covariance of the most abundant microbial species of the preterm infant gut. The force-driven network was constructed using bacterial taxa as nodes and covariances as edges. Red edges correspond to negative correlations, while green edges represent positive associations. The node size is proportional to the degree of interactions, while node colors represent the 13 obtained clusters of covariating species.

To assess the ecological role of the low-abundance taxa in the preterm infant gut, we explored the microbial communities structure using cooccurrence network analysis (Table S6). Modularity analysis revealed the presence of 13 clusters of covariating species, which were highlighted with different node colors (Fig. 3). As depicted in Figure 3, the five microbial species predominant in the PT-CSTs1-5 correspond to the higher interconnected nodes, suggesting that they are also more linked with other infant gut microbiota members. Furthermore, most of the analyzed bacterial taxa are engaged in widespread negative relationships, while the positive ones were observed among minority members of the preterm infant gut, which give rise to a web of interactions supporting the growth of the predominant taxa (Fig. 3) (43).

For example, dominant species of PT-CSTs, such as S. agalactiae (node 156), E. coli (node 151), E. faecalis (node 150), and K. pneumoniae (node 148), were engaged in mutual relationships with known low-abundance members of the preterm infant gut microbiota, including *Streptococcus*, *Cutibacterium*, *Enterobacter*, and *Corynebacterium* genera (green group), as well as members of the *Actinobacteria* family (violet group) (Fig. 3). This finding suggests that these specific minor players of the bacterial population can markedly shape the infant gut microbiota community by playing a potentially crucial role in sustaining the balance of the interaction network. Thus, a loss of marginal species could drive the disruption of the intricate microbial network, allowing for potential persistent colonization by (opportunistic) pathogens. Overall, these network analyses provide an overview of the bacterial interactions underlying the predominance of the taxa observed in PT-CSTs, adding new dimensions to our understanding of gut dysbiosis in neonates affected by NEC.

### Assessing of gut microbial metabolic pathways in NEC and healthy subjects.

As mentioned above, the gastrointestinal microbial inhabitants may influence host health through their metabolic activities, which participate in biomodification or *de novo* synthesis of metabolites. Therefore, changes in the microbiome gene repertoire, reflecting shifts in the gut microbiota composition, were analyzed to allow us to understand how potential gut bacterial community-derived metabolites differ between NEC patients and healthy subjects, with particular focus on essential reactions of metabolic pathways, as reported by literature and the MetaCyc database (44) (Table S7). Moreover, potential association between gut-associated bacterial communities and the microbial metabolic pathways was assessed by a Pearson correlation analysis. Specifically, a Pearson coefficient was calculated for each species and each microbial enzyme to determine whether they were significantly increased or decreased in NEC subjects compared to those in healthy infants (Table S8).

**FIG 4:**
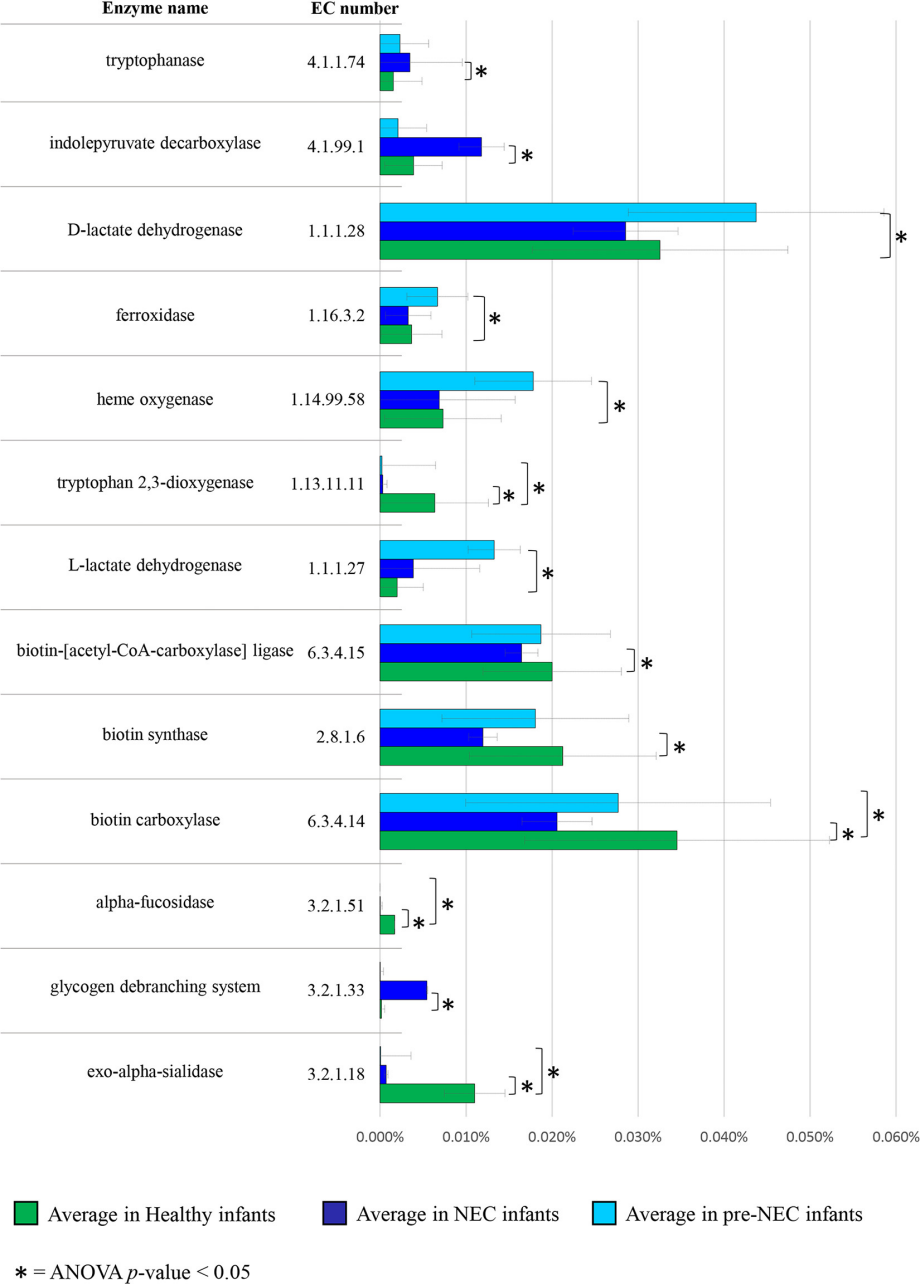
Statistically significant enzymes differentially encoded by gut microbiota of healthy, NEC, and pre-NEC infants. Bar plot depicts the relative average abundance of each considered enzyme in healthy, NEC, and pre-NEC samples on the horizontal axis. The table on the left reports the corresponding EC numbers and enzyme names.

In particular, in healthy infants, we detected enzymes related to glycosylated proteins degradation, i.e., α-fucosidase (EC 3.2.1.51) and sialidase (EC 3.2.1.18), that were almost entirely absent in NEC microbiomes (Table S7; Fig. 4). These enzymes are essential for releasing l-fucose and sialic acid from host-derived glycans such as intestinal mucins and human milk oligosaccharides (HMOs). Indeed, they are typically associated with gut commensals with health-promoting properties that coevolved strictly with the human host, such as Bifidobacterium longum, Bifidobacterium breve, and Bifidobacterium bifidum (43, 45–47). Moreover, the covariance analysis highlighted that α-fucosidase and sialidase were positively associated with members of *Blautia*, *Cutibacterium*, and *Enterobacter* genera and negatively with E. coli (Table S8).

Among the bacterial tryptophan degradation pathways, the key enzymes tryptophanase (TnaA, EC 4.1.99.1) and indolepyruvate decarboxylase (EC 4.1.1.74), catalyzing the production of indole and indole 3-acetic acid (IAA) biosynthesis, respectively, showed a 3-fold increase in NEC subjects compared to those in healthy infants (ANOVA *P* value of <0.05) (Table S7; Fig. 4). Notably, as confirmed by positive correlation index reported in Table S8, this may be the result of a higher abundance in NEC individuals of mainly E. coli, as well as members of *Klebsiella* and *Staphylococcus* genera, reported to produce these enzymes (48). Although these metabolites are known to have a role in regulating intestinal immunity acting as aryl hydrocarbon receptor (AhR) ligands, their effects are subjected to dietary tryptophan intestinal availability. The broad gastrointestinal damages experienced by NEC infants interfere largely with tryptophan assimilation, likely resulting in tryptophan depletion at the gut level and, therefore, lack of its microbial metabolization.

Although marginally, a portion of tryptophan (TRP) may be metabolized through the kynurenine pathway (KP), whose downstream metabolites, such as kynurenine (Kyn), quinolinic acid, picolinic acid, and kynurenic acid (KA), are known for their neuroactive properties, also regulating various (bio) processes related to inflammation and immune response (49–52). The enzyme tryptophan 2,3-dioxygenase (TDO; EC 1.13.11.11), catalyzing the first and rate-limiting step of the KP, was detected with a 4-fold increase in healthy subjects (ANOVA *P* value of <0.05) (Table S7; Fig. 4). As suggested previously, although TDO is typically a eukaryotic enzyme, these results supported the notion that some bacteria could synthesize Kyn by expressing an enzyme homologous to TDO (53), thus suggesting that depletion of bacterial homologous for this enzyme in NEC subjects may reflect increased gut inflammation and/or unbalanced intestinal mucosal reactivity (50, 54). Remarkably, positive associations were found between TDO abundance and the presence of various commensals of the infant gut, including *Bacteroides*, *Cutibacterium*, and *Enterobacter* genera, while negative correlations were identified with E. coli and S. epidermidis. Furthermore, biotin (vitamin B7) metabolism-related enzymes, including biotin-(acetyl coenzyme A [acetyl-CoA] carboxylase) ligase (EC 6.3.4.15), biotin carboxylase (EC 6.3.4.14), and biotin synthase (EC 2.8.1.6), which catalyzes the essential reaction in the biotin biosynthetic pathway, were found to be down-represented in NEC patients decreased, respectively, by 18.58%, 40.64%, and 43.85% compared to those in healthy samples (ANOVA, *P* < 0.05) (Table S7; Fig. 4). Interestingly, biotin has a putative effect on a range of catabolic and anabolic pathways of the human host, such as carbohydrates and amino acids catabolism or fatty acids synthesis (54), and it can be synthesized at the intestinal level by gut commensals possessing the complete biosynthesis pathway, such as Bacteroides fragilis, which is a bacterial taxon enriched in healthy infants (55). Furthermore, negative correlation coefficient was observed between components of biotin biosynthetic pathway and E. coli (Table S8).

In contrast, among the microbial pathways overexpressed in the fecal samples of NEC patients, we found the glycogen debranching enzyme (EC 3.2.1.196) to be 10-fold higher than that of controls (ANOVA *P* value of <0.05) (Table S7; Fig. 4). This enzyme is essential to complete the glycogen breakdown through the glycogen metabolism pathways (56) and has been referred to as a potential virulence factor in many microorganisms. Indeed, enzymes involved in this complex carbohydrate catabolism might participate in pathogen infectivity, contributing to virulence, colonization, and the environmental survival of pathogen strains. This reflects the increased abundance of E. coli and other members of the *Gammaproteobacteria* class (57), such as *Pseudomonas* and *Klebsiella*, observed in NEC infants compared to that in healthy subjects (58–60).

### Gut microbial community analysis in pre-NEC samples.

**FIG 5:**
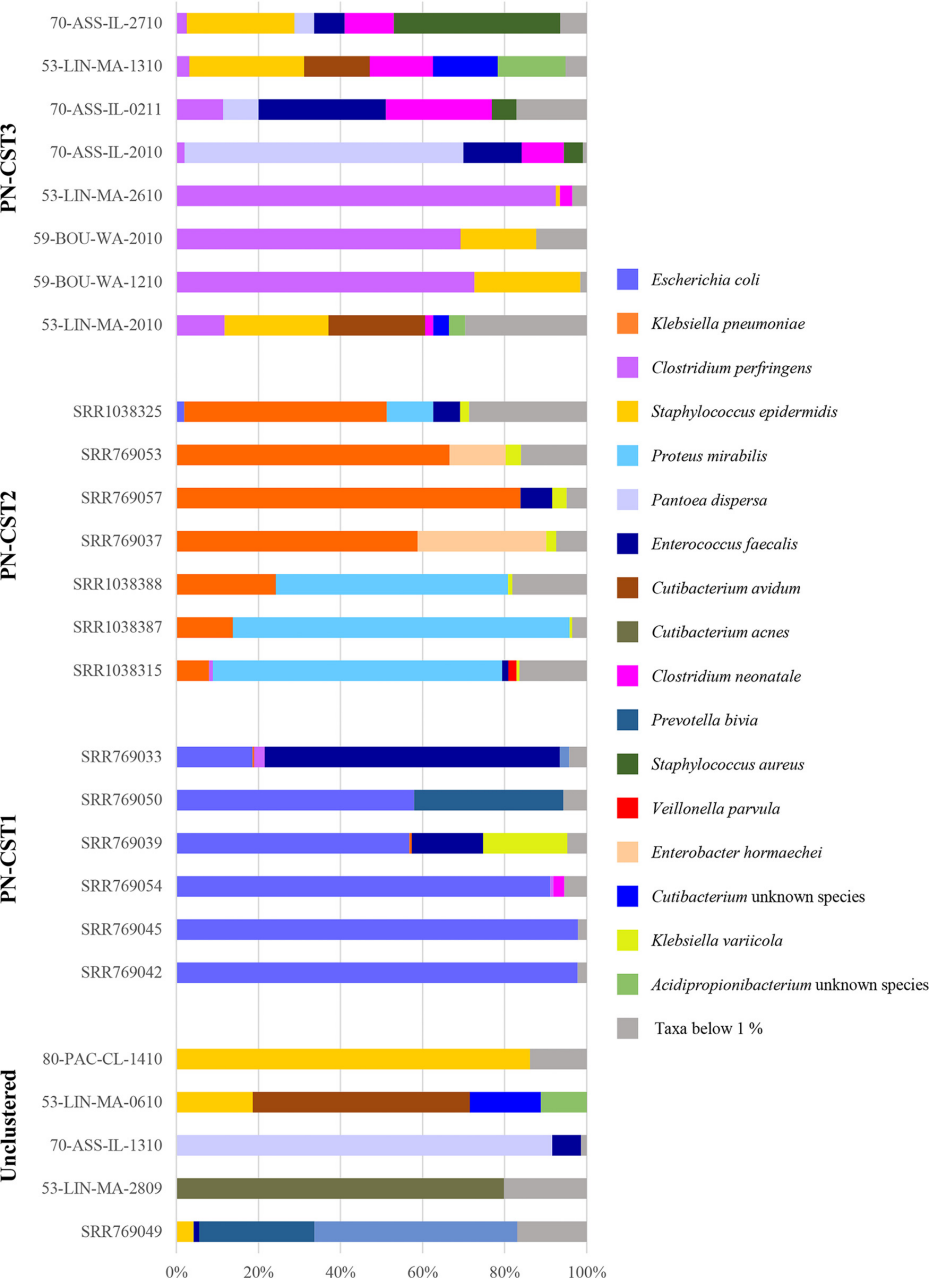
Species-level taxonomic composition of pre-NEC infants. Pre-NEC samples were grouped according to the specific PN-CST to which they belong. Relative abundances are reported on the vertical axis and the samples on the horizontal axis.

In order to identify possible microbial signatures causatively involved in NEC development, we compared the taxonomic profiles of 26 fecal samples collected from preterm infants before NEC development (pre-NEC) versus those from all 81 healthy controls. Focusing on the gut bacterial community composition of pre-NEC infants, HCA identified three distinct recurrent taxonomic profiles, named Pre-NEC community state types (PN-CST1 to 3), that could be observed in preterm infants who later developed NEC (Table S9; Fig. 5). Notably, each PN-CST appears to be dominated by one or few recognized (opportunistic) pathogens.

In particular, despite the fact that PN-CST1 seems to be dominated by common gut colonizers such as E. coli, E. faecalis, or members of the *Prevotella* genus, the two most abundant species observed in each profiled sample constitute >90% of the whole microbial population, thus indicating a marked simplification of the microbial population, i.e., reduced biodiversity (Table S9). In contrast, PN-CST2 and PN-CST3 showed higher taxonomic complexity. Still, they were dominated by well-recognized pathogens frequently involved in nosocomial infections, such as K. pneumoniae (61) and Proteus mirabilis (62, 63) in PN-CST2 or Clostridium perfringens (64), Clostridium neonatale (65), Pantoea dispersa (66, 67), and Staphylococcus aureus (68) in PN-CST3 (Table S9; Fig. 5). Remarkably, the latter pathogens are absent or present at a much lower relative abundance in healthy preterm infants who did not develop NEC (K. pneumoniae and P. mirabilis showed an average abundance of 43.49% and 31.51% in PN-CST2, respectively, in contrast to 3.83% and 0% in control, respectively; C. perfringens, C. neonatale, P. dispersa, and S. aureus displayed an average abundance of 26.52%, 6.85%, 8.14%, and 5.12% in PN-CST3, respectively, rather than 1.02%, 1.21%, 0%, and 0.65% in healthy subjects, respectively; *t* test *P* values of <0.01). In contrast, P. mirabilis, C. perfringens, *C. neonatale*, and *P. dispersa* were found to be almost absent also in NEC microbiomes (C. perfringens and *C. neonatale* showed an average abundance of 0.62% and 0.12%, respectively, while P. mirabilis and *P. dispersa* were absent), likely as a consequence of the broad-spectrum antibiotic treatments. This suggests that the above-mentioned taxa could represent early microbial signatures to be further investigated. Nevertheless, culture-based analyses of NEC microbiota should be performed to corroborate the relevance of microbial biomarkers as putative targets for noninvasive screening able to select infants with “high-risk” microbial patterns. Notably, this CST-based overview suggested that the development of NEC should be considered a multifactorial event that involves a loss in biodiversity accompanied by the overgrowth of specific opportunistic pathogens based on the taxonomic pattern initially established in each preterm infant.

In order to identify putative novel metabolic biomarkers of NEC that can support early diagnosis of this disease, we performed the prediction of microbial enzymatic reactions based on the shotgun metagenomics data of the 26 pre-NEC infant fecal samples. Interestingly, among the bacterial metabolites which were significantly increased before clinical development of NEC, we detected enzymes involved in iron uptake and heme degradation, i.e., ferroxidase (EC 1.16.3.2) and heme oxygenase (EC 1.14.99.58) (Table S10; Fig. 4), whose activities are essential for Gram-negative pathogens, which obtain the iron requested for their own growth from heme sequestered from their hosts (69, 70). In particular, such microbial enzymes showed an increase of 45% and 143% in the pre-NEC stage with respect to those in healthy controls (Table S10; Fig. 4). Furthermore, the metabolites profiling of gut bacteria also revealed that the enzymes l- and d-lactate dehydrogenase (EC 1.1.1.27 and EC 1.1.1.28, respectively) were more abundant in pre-NEC infants than in their healthy counterparts, showing a 373% and a 34% increase, respectively (*t* test *P* < 0.05) (Table S10; Fig. 5). In contrast, compared to the control group, infants who received NEC diagnosis did not show such a high abundance of dl-lactate dehydrogenase (*t* test *P* value of >0.05), likely due to the broad antibiotics treatments as well as invasive medical and surgical procedures leading to the disruption of the gut microbiota and hence of the lactate-producing bacteria (71). However, a recent study reported higher levels of lactate in the urine metabolome of preterm newborns with NEC compared to those of the control group, reinforcing the possible connection between this metabolite and NEC onset (72). In addition, dl-lactate has been reported to accumulate in feces from individuals with ulcerative colitis and inflammatory bowel disease (73, 74), highlighting the potential negative consequences of elevated intestinal lactate levels leading to gut acidosis due to its pH lowering effect (75, 76). Moreover, it has been demonstrated that lactate accumulation may occur due to a decrease in lactate-utilizing bacteria, such as members of *Bacteroidetes* and *Firmicutes* phyla (76–78). These observations suggested that an imbalance between gut communities of lactate-producing and lactate-utilizing bacteria leading to gastrointestinal dl-lactate accumulation can arise in infants who later develop NEC. Thus, it can represent a possible predictive biomarker of NEC, although future experimental studies employing analytical methods are needed to validate the clinical value of lactate level in infant stool at the pre-NEC stage.

### Conclusion.

Necrotizing enterocolitis (NEC) is a relatively common severe gastrointestinal disease affecting mainly low-birth-weight infants, with a reported mortality rate ranging between 15% and 30% (79). Recently, the increased prevalence of this intestinal disease has been linked mainly with early gut microbial dysbiosis and dysregulated immune response leading to overemphasized gastrointestinal inflammation in preterm infants.

In order to explore the gut microbial communities before and during NEC, we performed one of the largest shotgun metagenomics meta-analyses, including 124 publicly available data sets of preterm infant fecal samples along with 47 shotgun metagenomics data sets obtained from fecal samples of 18 preterm infants collected across the first month of life in the framework of this study at the NICU Croix Rousse University Hospital. Statistically validated hierarchical clustering analysis allowed the identification of five archetypical recurring taxonomic profiles named preterm community state types encompassing both healthy controls and NEC-affected preterm infants. Investigation of the NEC subpopulation of each PT-CST revealed a common trend of biodiversity loss accompanied by a marked increase in renowned (opportunistic) pathogens. Moreover, reduction in biodiversity reflected the loss of commensal or protective/health-promoting taxa following NEC development, including members of the genera *Bifidobacterium* and *Akkermansia*. The ecological role of minor components of the gut microbial population of preterm infants was further explored by covariance analysis. The resulting force-driven network revealed that specific clusters of microorganisms, constituted by both dominant and low-abundance taxa, tend to covariate, thus indicating an intricate network of ecological relationships explaining how the loss of minor players is responsible for such broad effects on the whole taxonomic profile. Overall, this CST-based overview of taxonomic features associated with NEC patients or healthy preterm infants revealed that biodiversity reduction driven by the loss of specific taxa might represent valuable microbial biomarkers for the early diagnosis of NEC and a starting point for the development of novel bacteria-based therapies. However, the clinical significance of these possible translational outcomes will need further validation in follow-up clinical trials.

The shotgun metagenomics data were also employed for the investigation of the occurrence of metabolic pathways. Intriguingly, we revealed that enzymes involved in tryptophan metabolism, biotin synthesis, and HMOs degradation were depleted in samples of preterm infants diagnosed with NEC compared to those in healthy controls, while the enzyme lactate dehydrogenase (LDH) was overabundant in preterm infants prior to NEC development. This suggests a gastrointestinal dl-lactate accumulation arising in infants who later develop NEC, making this compound a potential functional biomarker for early diagnosis of NEC.

## MATERIALS AND METHODS

### Ethical statement.

The human study protocol was approved by the local Ethics Committee (Comité de Protection des Personnes Sud-Est IV, Lyon).

### Data collection.

To perform a meta-analysis, we retrieved four publicly available data sets from studies involving the taxonomic evaluation of the gut microbiota in preterm infants with and without NEC (PRJNA46337, PRJNA376566, PRJNA63661, and PRJNA273761). Remarkably, we selected shotgun metagenomics data sets achieved by the Illumina sequencing platform to obtain high resolution and limited input data variability. Accordingly, we collected 124 fecal samples from five different neonatal intensive care units (NICU) in the United Stated, corresponding to 57 infants considered overall healthy and 67 declared affected by NEC.

Additional 47 fecal samples from 18 premature infants enrolled in NICU at Croix Rousse University Hospital of Lyon, France, were considered between September 2014 and November 2014. Specifically, fecal samples of infants were collected weekly during their first 40-day NICU stay. Seven infants developed NEC (stage II and stage III NEC diagnosis) (80), while 11 with no infectious intestinal complications have been regarded as controls.

### Sample collection and DNA extraction.

Stool samples were kept on ice immediately after collection and shipped to the laboratory under frozen conditions, where they were preserved at −20°C until they were processed. DNA extraction from each sample was performed using the QIAmp DNA stool minikit following the manufacturer’s instructions (Qiagen, Germany). DNA concentration and purity were determined employing a Picodrop microliter spectrophotometer (Picodrop).

### Sequencing and taxonomic classification.

The shotgun metagenomic sequencing was performed by GenProbio Srl (http://www.genprobio.com/). DNA library preparation was performed using the Nextera XT DNA sample preparation kit (Illumina, San Diego, CA), following the manufacturer’s instructions. One nanogram input DNA from each sample was used for library preparation. The isolated DNA underwent fragmentation, adapter ligation, and amplification. The ready-to-go libraries were pooled equimolarly, denatured, and diluted to a sequencing concentration of 1.5 pM. Sequencing was performed on NextSeq 550 instrument (Illumina, San Diego, CA), following the manufacturer’s instructions, using the 2 by 150 bp high output sequencing kit and spike-in of 1% PhiX control library. Shotgun metagenomics analysis of the fecal samples produced an average number of 3,314,112.39 ± 6,556,669.69 reads per sample. The raw data in fastq format, including those retrieved from publicly available shotgun data sets, were submitted to quality filtering for the removal of reads with an average quality of <25. Subsequently, human DNA was removed by reads mapping on the Homo sapiens genome, obtaining an average of 13,891.71 ± 3,580.60 reads per sample that were submitted to downstream analysis.

Following that, taxonomic profiling of retained reads was performed with the METAnnotatorX bioinformatics platform (25). Remarkably, in order to homogenize the sequencing data from different studies, we selected only those produced through Illumina sequencing method. Taxonomic classification of each sequenced read was achieved using MegaBLAST (81), employing the curated nonredundant sequence database of genomes retrieved from the National Center for Biotechnology Information (NCBI). For each metagenomic sample, species richness, i.e., biodiversity, represented the number of gut-associated bacterial taxa whose sequenced reads had a relative abundance greater than 0.5%. Similarities between samples (beta-diversity) were calculated by Bray-Curtis dissimilarity based on species abundance. The range of similarities is calculated between values 0 and 1. Principle-coordinate analysis (PCoA) representation of beta-diversity was performed using ORIGIN 2021 (https://www.originlab.com/2021). In the PCoA, each dot represented a sample distributed in tridimensional space according to its own bacterial composition.

### Functional prediction.

Functional profiling of sequenced reads was obtained through the METAnnotatorX bioinformatics platform (25). In addition, enzymatic reactions profiles were predicted using METAnnotatorX based on the MetaCyc database (82).

### Microbial cooccurrence and network analyses.

Covariance analysis involving the 233 bacterial species obtained by shotgun profiling of the 171 infant fecal samples was accomplished employing Kendall’s tau rank covariance analysis (83). Using software Gephi (https://www.gephi.org/), such correlation coefficients were then exploited to build a force-driven network, where bacterial species, represented in the form of nodes, are connected by edges. Each node size is proportional to the number of interactions of a specific taxon, i.e., the node degree, while the edge color indicates the type of interaction, i.e., positive (green) or negative (red).

### Statistical analyses.

Sample clustering based on different predominant taxa was achieved by hierarchical clustering analysis (HCL) using bacterial abundance information at the species level and was then calculated through TMeV 4.8.1 software using Pearson correlation as a distance metric. The data obtained were represented in the form of a cladogram. ORIGIN 2021 (https://www.originlab.com/2021) and the online version of Medcalc software (https://www.medcalc.org/) were used to compute statistical analyses, including *t* test, ANOVA, and chi-square test. PERMANOVA was performed using 1,000 permutations to estimate *P* values for differences among populations in PCoA. Furthermore, the differential abundance of bacterial genera was tested by *t* test. Covariance analysis between microbial community and bacterial metabolic pathways was obtained through a Pearson correlation analysis between the abundances of the species and abundances of each individual enzyme observed in the profiled data sets.

### Data availability.

Shotgun metagenomics data have been deposited in the NCBI Short Read Archive (SRA) under BioProject code PRJNA733860.
