# Supplementary material for: Unraveling the Microbiome of Necrotizing Enterocolitis: Insights in Novel Microbial and Metabolomic Biomarkers
Source: Microbiol Spectr. 2021 Oct 27;9(2):e01176-21. doi: 10.1128/Spectrum.01176-21 (PMC8549755; doi:10.1128/Spectrum.01176-21)
Supplement: SUPPLEMENTAL FILE 2 — Supplemental material. Download SPECTRUM01176-21_Supp_2_seq10.pdf, PDF file, 0.3 MB [file spectrum01176-21_supp_2_seq10.pdf]

Unraveling the microbiome of necrotizing enterocolitis: insights in novel microbial and metabolomic biomarkers

Running title: Potential microbial biomarkers in necrotizing enterocolitis

Key words: necrotizing enterocolitis, NEC, microbiota, metagenomics, shotgun

Chiara Tarracchini[1§], Christian Milani[1,2§], Giulia Longhi[1,3], Federico Fontana[1,3], Leonardo Mancabelli[1], Roberta Pintus[4], Gabriele Andrea Lugli[1], Giulia Alessandri[1], Rosaria Anzalone[3], Alice Viappiani[3], Francesca Turroni[1,2], Michele Mussap[5], Angelica Dessì[4], Flaminia Cesare Marincola[6], Antonio Noto[7], Anna De Magistris[4], Marine Vincent[8], Sergio Bernasconi[2], Jean-Charles Picaud[8], Vassilios Fanos[4,5#], Marco Ventura[1,2#]

§ These authors contributed equally

# These authors contributed equally

Laboratory of Probiogenomics, Department of Chemistry, Life Sciences, and Environmental Sustainability, University of Parma, Parma, Italy[1]; Microbiome Research Hub, University of Parma, Parma, Italy[2]; GenProbio Srl, Parma, Italy[3]; Neonatal Intensive Care Unit, Department of Surgical Sciences, University of Cagliari, Monserrato, Italy[4] ; Neonatal Intensive Care Unit, AOU, Cagliari, Cagliari, Italy[5]; Department of Chemical and Geological Sciences, University of Cagliari, Monserrato, Cagliari, Italy[6]; Department of Medical Science and Public Health, University of Cagliari, Monserrato, Cagliari, Italy[7]; Neonatology Unit, Croix-Rousse University Hospital, Hospices Civils de Lyon, Lyon, France and and CarMen laboratory, INSERM, INRA, Claude Bernard university Lyon1, Pierre Benite, France[8]

# Correspondence

Mailing address for Marco Ventura, Department of Life Sciences, University of Parma, Parco Area delle Scienze 11a, 43124 Parma, Italy. Phone: ++39-521-905666. Fax: ++39-521-905604. E-mail: marco.ventura@unipr.it

Mailing address for Vassilios Fanos, Neonatal Intensive Care Unit, Department of Surgical Sciences, AOU and University of Cagliari, Monserrato, Italy, vafanos@tin.it

Supplementary Text

Composition and structure of the Pre-Term Community State Types (PT-CSTs).

Hierarchical Clustering Analysis (HCA) based on the species-level microbial profiling allowed the identification of five archetypal subgroups named Pre-Term Community State Types (PT-CSTs) (Table S4). In detail, PT-CST1 is composed mainly (75 %) by subjects classified as NEC. Specifically, PT-CST1 (n = 12) was characterized by a high abundance of *E. faecalis* (average of 59.44 %) with a prevalence of 100 %. Interestingly, the NEC subjects belonging to this subgroup showed higher average abundance of *Enterococcus faecalis* rather than Control samples (72.67 % vs 19.73 %, T-test *p*-value < 0.01) with concomitant presence of *Staphylococcus epidermidis* (Table S4, Figure 1b). In contrast, members of *Actinomyces*, *Corynebacterium*, *Schaalia*, and *Bifidobacterium*, which together constituted an average of about 52. 19 % of the healthy microbiome, were undetected in NEC samples (Table S5, Figure 2). While *St. epidermidis* commonly inhabits the human skin microbiota, in premature and very low bird weight infants this species can represent a bacterial agent causing neonatal sepsis and other neonatal morbidities, such as brain injury as well as pulmonary epithelial damage and was previously reported in NEC cases (1–4). Similarly, although *E. faecalis* was commonly found among early colonizers of the neonatal gut and, in certain circumstances, it can elicit biofilm-associated opportunistic infections, leading to clinical manifestations such as urinary tract infections, endocarditis, peritonitis, and NEC (5–8). Furthermore, acquired and natural antimicrobial resistance has been found among members of *Enterococcus* genus, likely providing *E. faecalis* with a biological advantage that allows it to defeat its niche competitors (5). Interestingly, the association of *S. epidermidis* and *E faecalis* appeared almost absent in preterm control newborns, thus emerging as possible NEC-associated biomarkers.

Moving the focus on other HCL-based clusters, PT-CST2, PT-CST4, and PT-CST5 were composed of both NEC and healthy subjects and were dominated by *Escherichia coli* (PT-

CST2), *S. epidermidis* (PT-CST4) or *Klebsiella pneumoniae* (PT-CST5) (Table S4, Figure 1b). Nevertheless, these three species showed statistically significant difference in intra-cluster abundance between NEC and healthy subjects. In particular, the 19 NEC cases of PT-CST2 showed a higher abundance of *E. coli* species (average of 86.51 %) in contrast to that found among the 14 healthy samples (67.44 %, T-test p-value < 0.05) (Table S4), accompanied by lower species richness than their healthy counterparts (average of 4 vs 7, T-test p-value < 0.05), with significantly reduction in *Bacteroides* genus members (T-test p-value < 0.05) (Table S4). In a similar fashion, the subjects with NEC of PT-CST5 exhibited a significantly higher abundance of *K. pneumoniae* compared to healthy subjects (66.54% vs 39.15 %, T-test p-value < 0.05) (Table S4) associated with a decreased biodiversity (9.6 vs 15.2 bacterial species per samples, T-test p-value < 0.05) and the lack of particular healthy infant gut-associated bacteria such as *Akkermansia* and *Bifidobacterium breve* taxa (9,10) (Table S5). Moreover, within PT-CST4 (n=18), showing *St. epidermidis* as dominant taxon with an average abundance of 78.33 %, NEC samples exhibited higher relative abundance of the abovementioned species (87.95 % compared to 72.22 % of the 11 Control samples, T-test p-value < 0.05) coupled with biodiversity loss (average of 5.4 vs 7.5 species per samples in healthy infants, T-test p-value < 0.05) (Table S4). Consistent with previous studies in preterm infants, these findings highlighted that although strains of *E. coli*, *K. pneumoniae*, and *St. epidermidis* were also detected in premature infants who do not develop NEC, there is a possible association between high intestinal levels of these taxa and NEC pathogenesis, specifically in a framework of biodiversity loss (11,12). In contrast, in PT-CST3 18 fecal samples from healthy subjects were combined with three NEC-affected. This subgroup was predominated by *S. agalactiae*, with an average abundance of 70.72 % and a prevalence of 66.6 % (Table S4, Figure 1b). Previous studies about *Streptococcus agalactiae* pathogenicity have reported that this microorganism can lead to neonatal infections with considerable variability in the degree of severity,

depending on the capsular serotype and strain-specific expression levels of other virulence factors. Thus, providing a possible explanation of the reasons, in this instance, supporting the identification of *Str. agalactiae* in the gut microbiota of healthy subjects (13,14)

Overall, these findings highlighted how NEC patients often experience a marked form of dysbiosis, where uncontrolled growth of a single bacterial species occurs with concomitant severe loss of biodiversity and/or particular microorganisms. This event eventually results in a permanent loss of competition between bacterial species that, in normal condition, leads to the early-stage development process of healthy human gut microbiota (15).

References

1. Moles L, Gómez M, Moroder E, Bustos G, Melgar A, del Campo R, et al. Staphylococcus epidermidis in feedings and feces of preterm neonates. PLoS One [Internet]. 2020 Feb 1 [cited 2021 Apr 30];15(2). Available from: https://pubmed.ncbi.nlm.nih.gov/32012172/
2. Albehair MA, Alosail MA, Albulwi NM, AlAssiry A, Alzahrani FA, Bukhamsin A, et al. A Retrospective Study on the Avoidability of Ventriculoperitoneal Shunt Infections in a University Hospital in Al-Khobar, Saudi Arabia. Cureus [Internet]. 2021 Feb 4 [cited 2021 Apr 30];13(2). Available from: https://pubmed.ncbi.nlm.nih.gov/33643750/
3. Friedrich AW. Control of hospital acquired infections and antimicrobial resistance in Europe: the way to go. Wiener Medizinische Wochenschrift [Internet]. 2019 Feb 1 [cited 2021 Apr 30];169(Suppl 1):25–30. Available from: https://pubmed.ncbi.nlm.nih.gov/30623278/
4. Both A, Huang J, Qi M, Lausmann C, Weißelberg S, Büttner H, et al. Distinct clonal lineages and within-host diversification shape invasive staphylococcus epidermidis populations. PLoS Pathog [Internet]. 2021 Feb 5 [cited 2021 Apr 30];17(2). Available from: https://pubmed.ncbi.nlm.nih.gov/33544760/
5. Pathogenesis and Models of Enterococcal Infection - PubMed [Internet]. [cited 2021 Apr 30]. Available from: https://pubmed.ncbi.nlm.nih.gov/24649512/
6. Ch'ng JH, Chong KKL, Lam LN, Wong JJ, Kline KA. Erratum to: Biofilm-associated infection by enterococci (Nature Reviews Microbiology, (2019), 17, 2, (82-94), 10.1038/s41579-018-0107-z) [Internet]. Vol. 17, Nature Reviews Microbiology. Nature Publishing Group; 2019 [cited 2021 Apr 30]. p. 124. Available from: https://pubmed.ncbi.nlm.nih.gov/30459455/
7. Goh HMS, Yong MHA, Chong KKL, Kline KA. Model systems for the study of Enterococcal colonization and infection [Internet]. Vol. 8, Virulence. Taylor and Francis

Inc.; 2017 [cited 2021 Apr 30]. p. 1525–62. Available from: https://pubmed.ncbi.nlm.nih.gov/28102784/

8. Delaplain PT, Bell BA, Wang J, Isani M, Zhang E, Gayer CP, et al. Effects of artificially introduced Enterococcus faecalis strains in experimental necrotizing enterocolitis. PLoS One [Internet]. 2019 Nov 1 [cited 2021 Apr 30];14(11). Available from: https://pubmed.ncbi.nlm.nih.gov/31675374/

9. Collado MC, Derrien M, Isolauri E, De Vos WM, Salminen S. Intestinal integrity and Akkermansia muciniphila, a mucin-degrading member of the intestinal microbiota present in infants, adults, and the elderly. Appl Environ Microbiol [Internet]. 2007 Dec [cited 2021 Apr 30];73(23):7767–70. Available from: https://pubmed.ncbi.nlm.nih.gov/17933936/

10. Mancabelli L, Tarracchini C, Milani C, Lugli GA, Fontana F, Turroni F, et al. Multi-population cohort meta-analysis of human intestinal microbiota in early life reveals the existence of infant community state types (ICSTs). Comput Struct Biotechnol J [Internet]. 2020 Jan 1 [cited 2021 Apr 30];18:2480–93. Available from: https://pubmed.ncbi.nlm.nih.gov/33005310/

11. Patel AL, Mutlu EA, Sun Y, Koenig L, Green S, Jakubowicz A, et al. Longitudinal survey of microbiota in hospitalized preterm very-low-birth-weight infants. J Pediatr Gastroenterol Nutr [Internet]. 2016 Feb 1 [cited 2021 Apr 30];62(2):292–303. Available from: https://pubmed.ncbi.nlm.nih.gov/26230901/

12. Dahl C, Stigum H, Valeur J, Iszatt N, Lenters V, Peddada S, et al. Preterm infants have distinct microbiomes not explained by mode of delivery, breastfeeding duration or antibiotic exposure. Int J Epidemiol [Internet]. 2018 Oct 1 [cited 2021 Apr 30];47(5):1658–69. Available from: https://pubmed.ncbi.nlm.nih.gov/29688458/

13. Manning SD, Ki M, Marrs CF, Kugeler KJ, Borchardt SM, Baker CJ, et al. The frequency of genes encoding three putative group B streptococcal virulence factors among invasive and colonizing isolates. BMC Infect Dis [Internet]. 2006 Jul 17 [cited 2021 Apr 30];6. Available from: https://pubmed.ncbi.nlm.nih.gov/16846499/

14. Bobadilla FJ, Novosak MG, Cortese IJ, Delgado OD, Laczeski ME. Prevalence, serotypes and virulence genes of Streptococcus agalactiae isolated from pregnant women with 35–37 weeks of gestation. BMC Infect Dis [Internet]. 2021 Dec 1 [cited 2021 Apr 30];21(1). Available from: https://pubmed.ncbi.nlm.nih.gov/33446117/

15. Aujoulat F, Roudière L, Picaud JC, Jacquot A, Filleron A, Neveu D, et al. Temporal dynamics of the very premature infant gut dominant microbiota. BMC Microbiol [Internet]. 2014 [cited 2021 Apr 30];14(1). Available from: https://pubmed.ncbi.nlm.nih.gov/25551282/

Supplementary Figure Legends

Figure S1. Exploration of the diversity and the gut microbiota composition of the pre-term infant. Panel a shows the box and whisker plot of the species richness observed in healthy, pre-NEC, and NEC infants. Panel b reports the principal coordinate analysis (PCoA) of the 171 collected pre-term infant samples.

a)

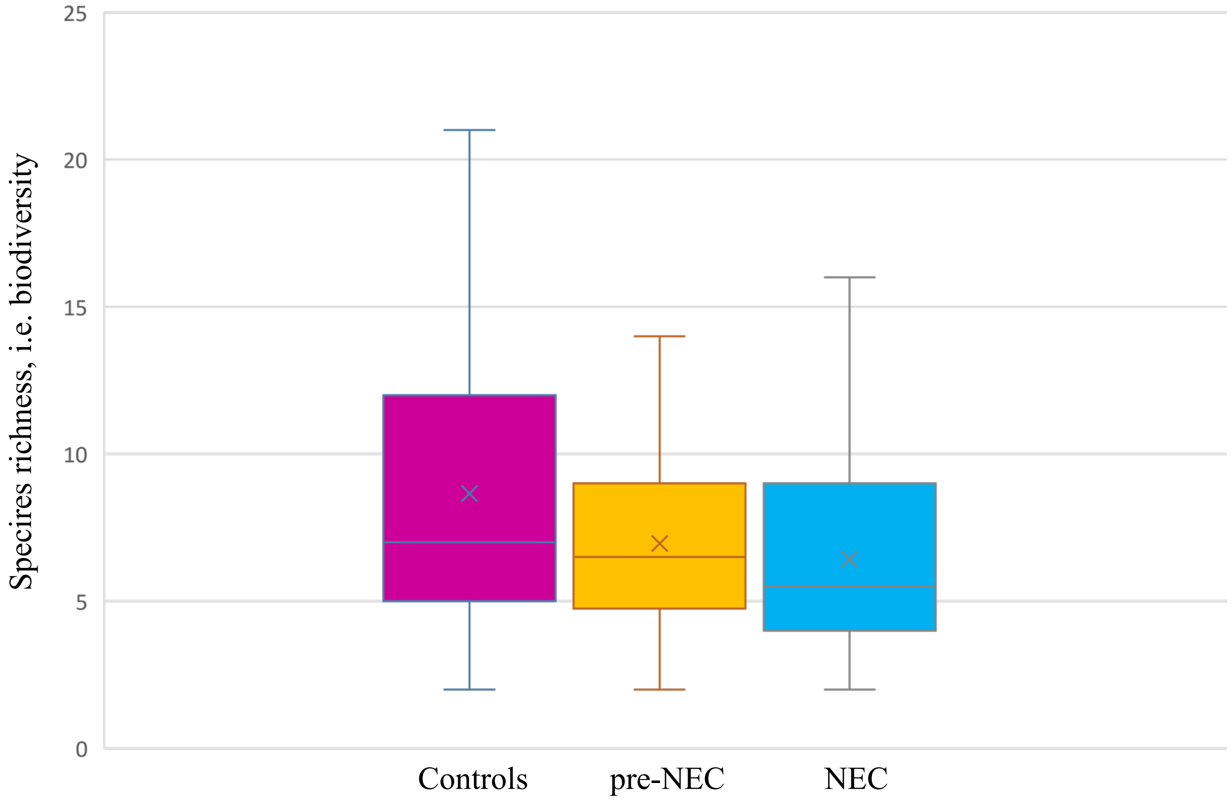

25
20
15
10
5
0
Specires richness, i.e. biodiversity
Controls
pre-NEC
NEC

b)

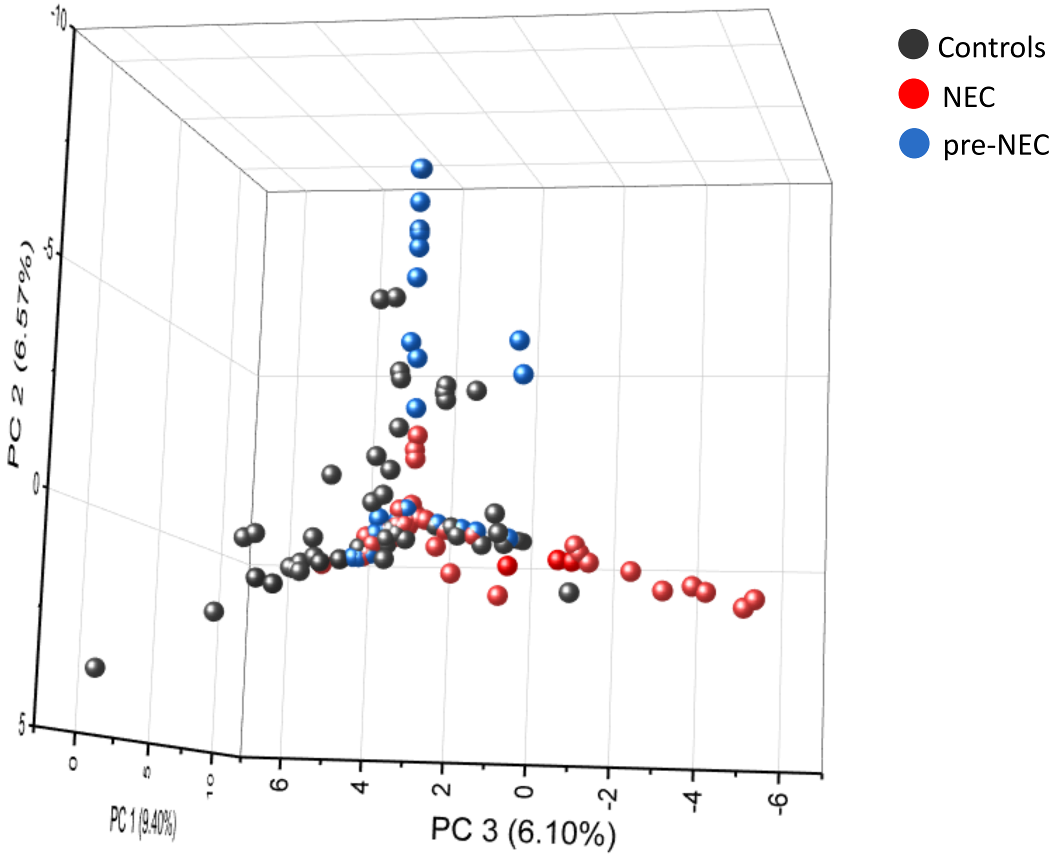

Controls
NEC
pre-NEC
PC 2 (6.57%)
PC 1 (9.40%)
PC 3 (6.10%)
6
4
2
0
-2
-4
-6

Figure S1
